# RAB35 is required for murine hippocampal development and functions by regulating neuronal cell distribution

**DOI:** 10.1038/s42003-023-04826-x

**Published:** 2023-04-21

**Authors:** Ikuko Maejima, Taichi Hara, Satoshi Tsukamoto, Hiroyuki Koizumi, Takeshi Kawauchi, Tomoko Akuzawa, Rika Hirai, Hisae Kobayashi, Inoya Isobe, Kazuo Emoto, Hidetaka Kosako, Ken Sato

**Affiliations:** 1 Laboratory of Molecular Traffic, Institute for Molecular and Cellular Regulation, Gunma University, Maebashi, Gunma 371-8512 Japan; 2 Laboratory of Food and Life Science, Faculty of Human Sciences, Waseda University, Tokorozawa, Saitama 359-1192 Japan; 3 Laboratory Animal and Genome Sciences Section, National Institutes for Quantum and Radiological Science and Technology, Chiba, Chiba 263-8555 Japan; 4 Department of Biological Sciences, School of Science, The University of Tokyo, Bunkyo-ku, Tokyo 113-0033 Japan; 5 Department of Molecular and Cellular Biology, School of Pharmaceutical Sciences, Ohu University, Koriyama, Fukushima 963-8611 Japan; 6 Department of Adaptive and Maladaptive Responses in Health and Diseases, Graduate School of Medicine, Kyoto University, Kyoto, 606-8507 Japan; 7 Division of Cell Signaling, Fujii Memorial Institute of Medical Sciences, Tokushima University, Tokushima, Tokushima 770-8503 Japan; 8 Gunma University Initiative for Advanced Research (GIAR), Gunma University, Maebashi, Gunma 371-8512 Japan

**Keywords:** Endocytosis, Neuronal development, Small GTPases

## Abstract

RAB35 is a multifunctional small GTPase that regulates endocytic recycling, cytoskeletal rearrangement, and cytokinesis. However, its physiological functions in mammalian development remain unclear. Here, we generated *Rab35*-knockout mice and found that RAB35 is essential for early embryogenesis. Interestingly, brain-specific *Rab35*-knockout mice displayed severe defects in hippocampal lamination owing to impaired distribution of pyramidal neurons, although defects in cerebral cortex formation were not evident. In addition, *Rab35*-knockout mice exhibited defects in spatial memory and anxiety-related behaviors. Quantitative proteomics indicated that the loss of RAB35 significantly affected the levels of other RAB proteins associated with endocytic trafficking, as well as some neural cell adhesion molecules, such as contactin-2. Collectively, our findings revealed that RAB35 is required for precise neuronal distribution in the developing hippocampus by regulating the expression of cell adhesion molecules, thereby influencing spatial memory.

## Abstract (web summary)

Mice with a brain-specific knockout of the Rab35 GTPase exhibit defects in spatial memory and hippocampal lamination, outlining a key role for Rab35 in hippocampal development and function.

## Introduction

The RAB proteins form the largest family of small GTPases that regulate intracellular membrane trafficking in eukaryotic cells^1^. Particularly, RAB35 is a highly conserved RAB protein in metazoans and is localized in the recycling endosome and plasma membrane. RAB35 was originally discovered as a regulator of endocytic recycling required for cytokinesis in HeLa cells^2,3^. In *Caenorhabditis elegans*, RAB35 controls the endocytic recycling of yolk receptors essential for oocyte growth and cell death^4–6^. *Drosophila* RAB35 functions in actin bundling, phagocytosis, neurotransmitter release, and spermatogenesis^7–10^. In mammals, RAB35 regulates several membrane trafficking pathways, including endosome-to-Golgi retrograde transport, phagocytosis, exosome secretion, and autophagy, in addition to its primary role in the recycling pathway^8,11–13^. Mammalian RAB35 also plays a role in multiple cellular processes associated with dynamic membrane changes, such as cytokinesis, immunological synapse formation, myoblast fusion, cilium length, and cell migration, in many kinds of culture cells and primary cells^2,14–18^. Additionally, previous studies in *Drosophila* and neuronal cell lines have reported several functions of RAB35 in the nervous system, including neurite outgrowth, axon elongation, exosome secretion, neurotransmitter release, oligodendrocyte differentiation, and synaptic vesicle turnover^9,11,19–22^. In fact, associations between RAB35 and neurodegenerative diseases, such as Parkinson’s disease and Alzheimer’s disease, have recently emerged^23,24^. Recently, RAB35 has also been identified as an oncogenic RAB that promotes cell proliferation^25^. However, the physiological importance of RAB35 in mammalian development remains largely unknown.

The formation of the mammalian brain is a highly orchestrated process that involves the generation, differentiation, migration, and maturation of neurons. Neuronal migration is an essential process to ensure the proper positioning of neurons and for the organization of multilayered structures, notably the cerebral cortex and hippocampus^26^. The impairment of this process has an adverse effect on neural networks and brain architecture, causing several neurological disorders, including lissencephaly, epilepsy, and schizophrenia^27–29^. The molecular mechanism associated with the formation of laminated structures has been primarily investigated in the cerebral cortex. Knockdown experiments based on in utero electroporation have revealed that RAB proteins related to endocytosis, such as RAB5, RAB7, RAB11, RAB18, and RAB23, regulate cortical neuronal migration in mice by controlling the traffic of N-cadherin during the distinct steps of endocytosis^30–32^. Although the formation process of the hippocampus is believed to be similar, its developmental mechanism, including the role of RAB proteins, is less well-understood.

To reveal the physiological function of RAB35 in mammals, here, we generated systemic *Rab35*-knockout mice and found that RAB35 is essential during early mouse embryogenesis. We also generated central nervous system (CNS)-specific *Rab35*-knockout mice and identified RAB35 as a requirement for proper hippocampal development. Our findings suggest that RAB35 plays an essential role in the proper positioning of pyramidal neurons in the hippocampus by regulating the expression of surface cell adhesion molecules, as well as being involved in appropriate spatial memory formation.

## Results

### RAB35 is essential for early mouse development

To investigate the physiological function of RAB35 in mammalian development, we generated *Rab35*-knockout mice using an ES cell line (Supplementary Fig. 1a). The disruption of the *Rab35* gene was validated using Southern blotting and PCR (Supplementary Fig. 1b, c). Heterozygous-targeted mice (*Rab35*^geo/+ or −/+^) were healthy and fertile. To obtain the homozygous knockout mice (*Rab35*^geo/geo^ and *Rab35*^−/−^), we intercrossed the heterozygous knockout and found that no offspring were either *Rab35*^geo/geo^ or *Rab35*^−/−^, indicating that *Rab35* null mice are embryonic lethal. At embryonic day (E) 15.5 and E11.5, no *Rab35* null embryos were obtained. At E9.5, 9 (32%) embryos were *Rab35* ^+/+^; 13 (46%) embryos were *Rab35*^−/+^; and 6 (21%) embryos were *Rab35*^−/−^ (Supplementary Fig. 1d). These results indicate that Rab35 is essential for early mouse development.

### RAB35 protein expression gradually increases during early brain development

**Fig. 1:**
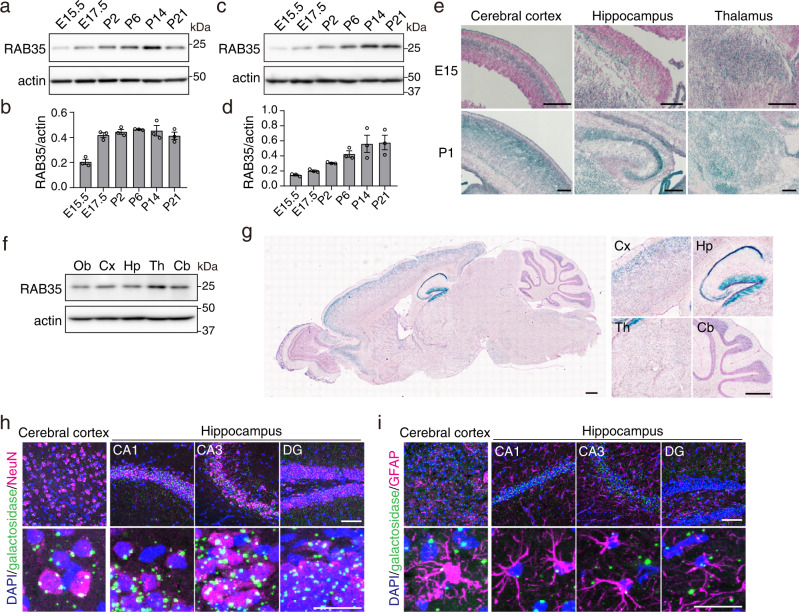
Expression profile of RAB35 in the mouse brain. **a** RAB35 protein levels in the whole mouse brain during the embryonic (E15.5 and E17.5) and postnatal (P2, P6, P14, and P21) periods. **b** Quantification of RAB35 protein levels in the whole mouse brain. Band intensities of RAB35 were normalized against those of actin (*n* = 3 per the indicated stage). **c** RAB35 protein levels in the hippocampus during the embryonic (E15.5 and E17.5) and postnatal (P2, P6, P14, and P21) periods. **d** Quantification of RAB35 protein levels in the mouse hippocampus (*n* = 3 per stage). **e** X-gal staining of sagittal sections from E15 and P1 *Rab35*^geo/+^ mouse brains. Scale bars, 200 μm. **f** RAB35 protein levels in 3-month-old mouse brain subregions. Ob olfactory bulb, Cx cerebral cortex, Hp hippocampus, Th thalamus, Cb cerebellum. **g** X-gal staining of a sagittal section of a 3-month-old *Rab35*^geo/+^ mouse brain. Scale bars, 500 μm. **h**, **i** Representative maximum intensity projection images of 4-month-old *Rab35*^geo/+^ mouse brain stained for β-galactosidase (green), NeuN or GFAP (magenta), and DAPI (blue). Scale bar; upper panel, 50 μm; lower panel, 20 μm. Data represent mean ± SEM.

Previous studies in *Drosophila* and neuronal cell lines have implied that RAB35 plays an important role in diverse neural functions, including neurotransmitter release, neurite elongation, oligodendrocyte differentiation, and exosome secretion^9,11,15,20^. We thus focused on the in vivo function of RAB35 in brain development. To this end, we first determined the expression profiles of RAB35 in the mouse brain. RAB35 protein levels gradually increased from E15 and peaked at postnatal day (P) 14 in the developing brain (Fig. 1a, b). Considering the whole brain, the RAB35 protein was expressed in the hippocampus at E15.5, with expression gradually increasing during development (Fig. 1c, d). LacZ staining detected RAB35 expression in several brain subregions, including the cerebral cortex, hippocampus, and thalamus at E15.5 (Fig. 1e). In the P1 brain, expression was more notable in the hippocampus and over a wide area of the cerebral cortex. In addition, RAB35 expression was identified in the olfactory bulb and cerebellum of adult mouse brains (Fig. 1f, g). Furthermore, we examined the cell types expressing RAB35 in adult mouse brains by immunostaining for β-galactosidase using either the mature neuronal marker NeuN or the astrocyte marker glial fibrillary acidic protein (GFAP). Maximum intensity projection images revealed β-galactosidase puncta in NeuN-positive and GFAP-positive cells, suggesting that RAB35 is expressed in neurons and astrocytes (Fig. 1h, i).

**Fig. 2:**
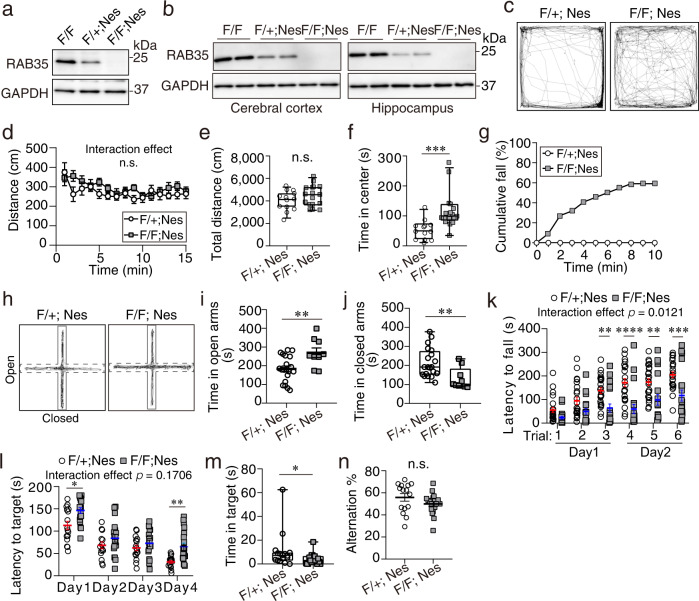
Characterization of CNS-specific *Rab35* conditional knockout mice. **a**, **b** Western blot analysis of E13.5 (**a**) and 4-month-old (**b**) *Rab35*^*flox/flox*^, *Rab35*^*flox/+*^; *Nestin-Cre*, and *Rab35*^*flox/flox*^; *Nestin-Cre* mouse brain lysates. **c**–**f** Open field test in control (*n* = 13) and *Rab35* cKO (*n* = 17) mice. **c** Typical traces of the horizontal locomotor activity. **d** Distance traveled per minute. Two-way repeated-measures ANOVA; interaction effect, *F*(14, 392) = 1.094, *p* = 0.3612. **e** Total distance traveled. Mann–Whitney test, *p* = 0.3. **f** Stay time in the center area. Mann–Whitney *U*-test, *p* < 0.001. **g**–**j** Elevated plus maze in control (*n* = 20) and *Rab35* cKO (*n* = 22) mice. **g** Cumulative percentage of mice falling from open arms. **h** Typical traces in the elevated plus maze test. **i** Stay time in open arms. Unpaired Student’s *t*-test, *p* = 0.0054. **j** Stay time in the closed arms. Mann–Whitney *U*-test, *p* = 0.0034. **k** Rotarod test in control (*n* = 23) and *Rab35* cKO (*n* = 20) mice. Two-way repeated-measures ANOVA with Bonferroni correction; interaction effect, *F*(5, 205) = 3.009, *p* = 0.0121; trial 3, *p* = 0.0076; trial 4, *p* < 0.0001; trial 5, *p* = 0.0059; trial 6, *p* = 0.0008. **l**, **m** Barnes maze tests in control (*n* = 16) and *Rab35* cKO (*n* = 19) mice. **l** Latency to reach the target hole. Each point represents the mean of three trials. Two-way repeated-measures ANOVA with Bonferroni correction; interaction effect, *F*(3, 99) = 1.706, *p* = 0.1706; day 1, *p* = 0.0261; day 4, *p* = 0.0030. **m** Time spent around the target hole during a 90 s period. Mann–Whitney *U*-test, *p* = 0.0136. **n** Alternation percentages in Y maze test of control (*n* = 15) and *Rab35* cKO (*n* = 18) mice. Unpaired Student’s *t*-test, *p* = 0.01454. Data represent mean ± SEM or box and whisker plots; box plot shows 25th and 75th percentiles (box), median (horizontal bar), and minimum to maximum (whiskers); n.s. not significant (*p* > 0.05); *p < 0.05; **p < 0.01; ***p < 0.001; and ****p < 0.0001.

To investigate the physiological importance of RAB35 in neural development, we generated CNS-specific *Rab35*-knockout mice by crossing mice harboring the *Rab35*^*flox*^ allele with transgenic mice expressing Cre recombinase under the control of the Nestin promoter (*Nestin-Cre*)^33^(Supplementary Fig. 1a). Although RAB35 protein expression was detectable in the mouse brain at E13.5 (Fig. 2a), it was undetectable in the whole brain of E13.5 *Rab35*^*flox/flox*^; *Nestin-Cre* embryos and in the cerebral cortex and the hippocampus of 4-month-old *Rab35*^*flox/flox*^; *Nestin-Cre* mice (Fig. 2a, b). CNS-specific *Rab35* conditional knockout (cKO) mice were born at the Mendelian frequency and were viable for more than 1 year. Male and female *Rab35* cKO mice gradually showed lower body weight than the control *Rab35*^*flox/+*^; *Nestin-Cre* mice. (Supplementary Fig. 1e, f).

### CNS-specific *Rab35* cKO mice exhibit reduced anxiety-related behaviors and motor function

To evaluate the impact of RAB35 deficiency on mouse behavior, we performed behavioral tests. In the open field test, which measures spontaneous motor activity and anxiety-related behavior in a novel environment, *Rab35* cKO mice exhibited spontaneous movement with travel times similar to the control mice (Fig. 2c–e), indicating that their horizontal locomotor activities were comparable. Meanwhile, the time spent in the center of the open field apparatus significantly increased in *Rab35* cKO mice (Fig. 2c, f). We then performed the elevated plus maze test to assess anxiety-related behaviors. Although 13 of the 22 *Rab35* cKO mice dropped from the open arms during the test (Fig. 2g), we evaluated the anxiety levels of nine mice that completed the test without falling. *Rab35* cKO mice spent significantly more time in the open arms and less time in the closed arms than control mice (Fig. 2h–j). These results indicated that *Rab35* cKO mice exhibit reduced anxiety-related behaviors.

In the accelerated rotarod test, which is used to assess motor function, control mice increased the time spent on the rotating rod after several trials, with most control mice eventually spending more than 150 s on a rod. In contrast, *Rab35* cKO mice exhibited a significantly reduced latency to fall (Fig. 2k). Taken together, these results suggest that RAB35 deficiency does not affect horizontal locomotor activity but causes defects in anxiety-related behaviors and motor function.

### *Rab35* cKO mice show a defect in spatial learning and memory

We further assessed spatial learning and memory using the Barnes maze test (Fig. 2l, m). The Barnes maze consists of a circular table with 16 holes around the perimeter and visual cues around the table. Of the 16 holes, only one contains an escape box underneath, considered the target. This test consists of several phases, including the acquisition phase and the probe test. During the acquisition phase, the spatial learning ability of each mouse was evaluated by measuring the time required to reach the target. As expected, the control mice learned the location of the target hole to avoid bright room lighting, with reduced time required to reach the target in each trial. *Rab35* cKO mice spent significantly more time reaching the target than control mice on day 4 (Fig. 2l). The probe test was conducted one day after the last acquisition trial to examine spatial memory acquisition. We removed the escape box from the table and measured the time spent in the proximity of the target to determine whether the mice had memorized the target hole position. As shown in Fig. 2m, *Rab35* cKO mice spent slightly but significantly less time around the correct target hole than control mice. To analyze the potential effects of RAB35 on short-term memory, mice were examined in a Y maze test. The percentage of spontaneous alternation in *Rab35* cKO mice was comparable with that in control mice (Fig. 2n). These results suggest that *Rab35* cKO mice exhibit deficits in spatial memory but not in working memory.

### The laminated structure of the cerebral cortex is preserved in *Rab35* cKO mice

Next, we investigated whether the loss of RAB35 affects brain architecture. The mammalian cerebral cortex has a six-layered structure. During cortical development, excitatory neurons are generated from radial glial cells in the ventricular side^34,35^. Newly born neurons radially migrate from the apical ventricular side toward the basal pial surface and stop migrating just beneath the marginal zone. Therefore, early-born neurons form the deep layer (layers VI and V), whereas late-born neurons pass through the deep layer and settle in more superficial layers (layers IV–II); hence, the proper six-layered structure of the cerebral cortex is formed in a birthdate-dependent inside-out manner^36,37^. Cortical neurons are segregated into cell layers with neurons formed around the same period and expressing layer-specific transcription factors, including chicken ovalbumin upstream promoter transcription factor-interacting proteins 2 (Ctip2; a marker for layer V) and cut homeobox 1 (Cux1; a marker for layer II–IV), to differentiate and establish layer-dependent neural connections.

**Fig. 3:**
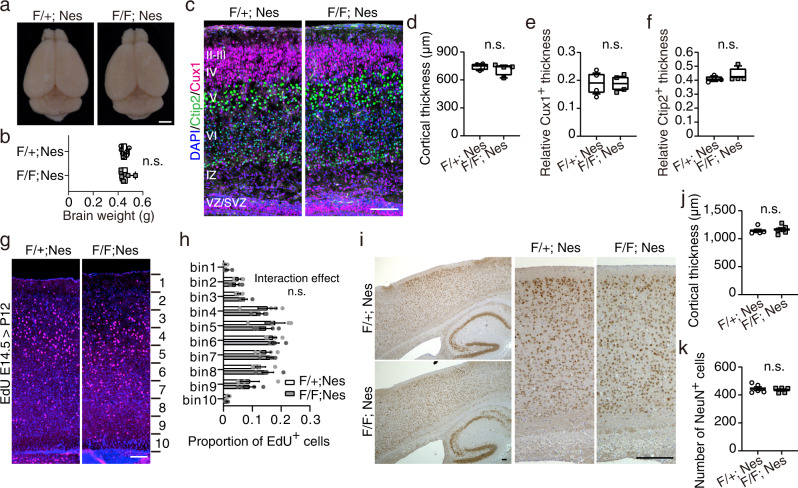
Laminated structure in the cerebral cortex is preserved in *Rab35* cKO mice. **a** Dorsal view of 3-month-old control and *Rab35* cKO mice brains. Scale bar, 2 mm. **b** Brain weights of 3-month-old control (*n* = 7) and *Rab35* cKO (*n* = 8) male mice. Mann–Whitney *U*-test, *p* = 0.8409. **c** Representative images of P0 cortices of the control and *Rab35* cKO mice immunostained for Cux1 (magenta), Ctip2 (green), and DAPI (blue). Scale bar, 100 μm. **d**–**f** Quantification of the cortical (**d**), relative Cux1-positive (**e**), and Ctip2-positive (**f**) layer thicknesses of P0 control (*n* = 4) and *Rab35* cKO (*n* = 4) mice. Cux1-positive and Ctip2-positive layer thicknesses were measured and normalized against total cortical thickness. Mann–Whitney *U*- test; **d**, *p* = 0.6857; **e**, *p*  >0.99; **f**, *p* = 0.2. **g** Representative EdU labeling birthdate analysis images of P12 cortex samples stained with EdU (magenta) and DAPI (blue). Scale bar, 100 μm. **h** Proportion of EdU-positive neurons in P12 cortices of control (*n* = 4) and *Rab35* cKO (*n* = 4) mice. Two-way repeated-measures ANOVA with Bonferroni correction; interaction effect, F(9, 54) = 0.4991, *p* = 0.8687. **i** Immunohistochemistry of sagittal sections of cerebral cortices of 4-month-old control and *Rab35* cKO mice using an anti-NeuN antibody. Scale bars, 100 μm. **j**, **k** Quantitative analysis of the cortical thickness (**j**) and the number of NeuN-positive cells in the 300-μm width cerebral cortex (**k**) of the control (*n* = 5) and *Rab35* cKO (*n* = 5) mice. Unpaired Student’s *t*-test; **j***, p* = 0.6928; **k**, *p* = 0.5375. Data represent mean ± SEM or box and whisker plots; box plot shows 25^th^ and 75^th^ percentiles (box), median (horizontal bar), and minimum to maximum (whiskers); n.s., not significant (*p* > 0.05).

The dorsal view or brain weight of *Rab35* cKO mice was comparable to that of control mice (Fig. 3a, b). In *Rab35* cKO mice, the thickness of the cerebral cortex and the relative thickness of the Cux1-positive superficial layer (layers II–IV), as well as that of the Ctip2-positive deep layer (layer V), were comparable with those of control mice at P0, the period when most of the cortical excitatory neurons reach their layer (Fig. 3c–f). We further evaluated neuronal migration in the cerebral cortex using the 5-ethynyl-2-deoxyuridine (EdU) labeling birthdate analysis (Fig. 3g, h). EdU was intraperitoneally injected into pregnant mice at E14.5, and the brains of the pups were collected at P12. The cerebral cortex was divided into 10 equal bins from the ventricular to pial surfaces, and the proportion of EdU-positive cells in each bin was quantified. RAB35-deficient cortical neurons labeled with EdU did not show significant migration defects. Immunostaining for NeuN revealed no significant difference in the distribution of NeuN-positive cells between the cortices of control and *Rab35* cKO adult mice (Fig. 3i). The thickness of the cerebral cortex and the number of NeuN-positive cells in the 300-μm wide cortices were also shown to be maintained in *Rab35* cKO mice (Fig. 3j, k). Thus, these results indicate that the laminated architecture is preserved in the cerebral cortex of RAB35-deficient mice.

### Loss of RAB35 induces heterotopic positioning of neurons in the hippocampus

**Fig. 4:**
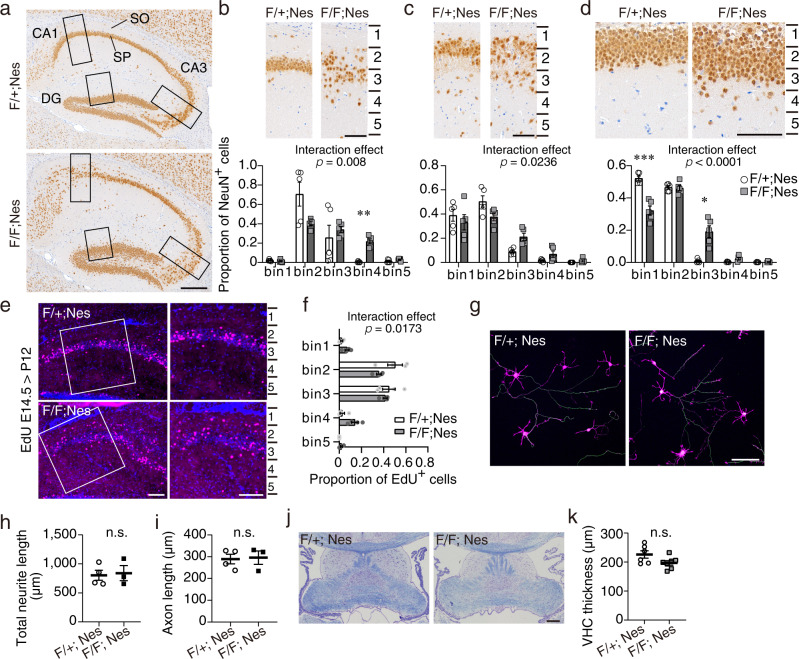
Ectopic distribution of principal neurons in *Rab35* cKO hippocampus. **a** Immunohistochemistry of sagittal sections of hippocampi of 4-month-old control and *Rab35* cKO mice using an anti-NeuN antibody. SP: stratum pyramidale, SO: stratum oriens. Scale bars, 200μm. **b**–**d** Distribution of NeuN-positive cells in the hippocampal CA1 (**b**), CA3 (**c**), and DG (**d**) regions of control (*n* = 4) and *Rab35* cKO (*n* = 4) mice. Two-way repeated-measures ANOVA with Bonferroni correction; **b** interaction effect, *F*(4, 32) = 4.161, *p* = 0.008; **c** interaction effect, *F*(4, 32) = 3.264, *p* = 0.0236; **d** interaction effect, *F*(4, 32) = 25.9, *p* < 0.0001. **e** Representative images of P12 hippocampi stained with EdU (magenta) and DAPI (blue). Scale bar, 100 μm. **f** Proportion of EdU-positive neurons in the P12 hippocampi of control (*n* = 4) and *Rab35* cKO (*n* = 4) mice. Two-way repeated-measures ANOVA with Bonferroni correction; interaction effect, *F*(4, 24) = 3.712, *p* = 0.0173. **g** Representative images of DIV 3 hippocampal primary neurons stained for p-NF (green) with rhodamine-phalloidin (magenta) and DAPI (blue). Scale bar, 100 μm. **h**, **i** Quantification of the rhodamine-phalloidin-positive total neurite length (**h**) and p-NF-positive axon length (**i**) of the control and *Rab35*-deficient primary neurons at DIV 3. At least 50 neurons from four (control) and three (*Rab35* cKO) different cultures were measured. Unpaired Student’s *t-*test; **h**, *p* = 0.8204; **i**, *p* = 0.8832. **j** Representative ventral hippocampal commissure (VHC) images of 3-month-old control and *Rab35* cKO mice (bregma −0.35 mm) by Klüver–Barrera staining. Scale bar, 200 μm. **k** VHC thickness in control (*n* = 6) and *Rab35* cKO (*n* = 7) mice. Unpaired Student’s *t*-test*, p* = 0.0695. Data represent mean ± SEM; n.s. not significant (*p* > 0.05); *p < 0.05; **p < 0.01; and ***p < 0.001.

In contrast with the cerebral cortex, we found that the laminated structure of the hippocampus was severely disorganized in *Rab35* cKO mice (Fig. 4a). The hippocampus contains a tightly packed pyramidal cell layer, also called stratum pyramidale (SP), of the Ammon’s horn (CA1, CA2, and CA3 regions) and a granule cell layer (GCL) of the dentate gyrus (DG) as shown in the control mice. In the *Rab35* cKO hippocampus, however, the SP in CA1 was remarkably fractured into stratum oriens (SO), and the pyramidal cells in CA3 were distributed more extensively. Ectopic pyramidal neurons were observed in the ventral hippocampal CA1 region (Supplementary Fig. 2a). Based on quantitative bin analysis, *Rab35* cKO mice exhibited significant changes in pyramidal cell distribution in CA1 and CA3 (Fig. 4b, c). The number of NeuN-positive neurons in either the RAB35-deficient CA1 or CA3 regions were comparable to those in controls (Supplementary Fig. 2b, c). Additionally, the granule cells in the DG dispersed towards the molecular layer with an increased cell number in *Rab35* cKO mice (Fig. 4d and Supplementary Fig. 2d). The CA1 neurons positive for Ctip2, here used as a postmitotic neuronal marker, presented a divided cell layer at P6, the period when most newly born hippocampal excitatory neurons arrive at their final position (Supplementary Fig. 2e), suggesting that hippocampal neurons ectopically position during the developmental stage.

### Loss of RAB35 leads to an impaired distribution of hippocampal pyramidal neurons

Since the rodent hippocampal pyramidal neurons in the CA1–CA3 regions are generated from a neuroepithelium dorsally shared with the neocortex, the laminated structures in the CA regions of the hippocampus are considered to form similar patterns to those in the cerebral cortex^27,38^. During the development of the cerebral cortex and the hippocampus, excitatory neurons are generated from two major types of progenitor cells: radial glial cells in the ventricular zone (VZ) and intermediate progenitors in the subventricular zone (SVZ)^34,35^. Radial glial cells produce intermediate progenitors or neurons in the VZ via asymmetric division, whereas intermediate progenitors migrate and accumulate in the subventricular zone (SVZ) just above the VZ. The formation of the laminated structures in the hippocampus primarily depends on the highly coordinated production and migration of neurons from the proliferative zones. The spatiotemporal control of neural progenitor cell proliferation is important for the production and positioning of neurons. In addition to proliferative potential, the morphology of radial glial cells also plays an important role in neuronal positions because newborn neurons use radial processes as scaffolds to migrate toward the final position in primitive SP.

We then investigated the functional and morphological integrity of the neural progenitors in the *Rab35* cKO hippocampus. The distributions of the paired box protein 6 (Pax6)-positive radial glial cells and T-box brain protein 2 (Tbr2)-positive intermediate progenitors were similar in the control and *Rab35* cKO CA regions at E15.5 (Supplementary Fig. 2f, g). The number of both progenitors was comparable in control and *Rab35* cKO mice (Supplementary Fig. 2h, i). Immunostaining for phosphorylated histone H3 (pHH3), a marker for cells undergoing mitosis, showed that the number of pHH3-positive cells in the *Rab35* cKO CA region did not change significantly compared with the control mice (Supplementary Fig. 2j, k). Similar results were obtained in DG (Supplementary Fig. 2l–n). These results indicate that the conditional deletion of *Rab35* using *Nestin-Cre* transgenic mice does not affect the spatial pattern and proliferation potency of neural progenitors in the hippocampus. Furthermore, we performed immunostaining using an antibody for Nestin, an intermediate filament protein in radial glial cells, to observe the morphology of the radial processes. Nestin-positive radial glial processes appeared to be normally organized in the sagittal sections of E15.5 *Rab35* cKO hippocampus (Supplementary Fig. 2o). From these data, the function and shape of the hippocampal neural progenitor cells are maintained in *Rab35* cKO mice.

Given that the loss of RAB35 did not impact neural progenitor cells, we examined whether malformation of the hippocampal layer could be attributed to defective migration or positioning of neurons during development using EdU labeling birthdate analysis (Fig. 4e, f). The hippocampal CA1 region, from the edge of the apical surface to 400 μm, was divided into five equal bins, and the proportion of EdU-positive cells in each bin was quantified. In control mice, over 90% of EdU-positive neurons reached bins 2 and 3, whereas, in *Rab35* cKO mice, the proportion of EdU-positive neurons in bins 2 and 3 decreased with an increase in bins 1 and 4 (Fig. 4f), thereby suggesting that neuronal cells could migrate, but were not distributed to the precise location. These findings indicate that RAB35 is required for the precise distribution of pyramidal cells in the developing hippocampus.

### *Rab35*-deficient hippocampal neurons exhibit superficially normal neuritogenesis

Based on previous studies that RAB35 knockdown suppresses the elongation of neurites in PC12 cells and axons in rat hippocampal primary neurons and the overexpression of wild-type or constitutively active mutant RAB35 enhances the elongation, we examined neuritogenesis in primary neurons harvested from the *Rab35* cKO mice. Hippocampal primary neurons cultured for days in vitro (DIV) 3 were subjected to immunostaining for an axon marker phosphorylated neurofilament heavy chain (pNF-H) and rhodamine-phalloidin (Fig. 4g). The lengths of both the phalloidin-positive total neurite and pNF-H-positive axon in RAB35-deficient primary neurons were comparable with those in control neurons (Fig. 4h, i), indicating that primary neurons derived from the *Rab35* cKO hippocampus sustain sufficient potential for neuritogenesis. To evaluate the role of RAB35 on axon elongation in vivo, we examined the thickness of the ventral hippocampal commissure (VHC) (Fig. 4j, k). *Rab35* cKO mice tended to exhibit a relatively reduced VHC thickness when compared with that of control mice; however, this difference was not statistically significant. These findings suggest that neuritogenesis is not markedly impaired despite the absence of RAB35.

### RAB35 is required for proper cell surface expression of neural cell adhesion molecules (NCAMs) in hippocampal neurons

**Fig. 5:**
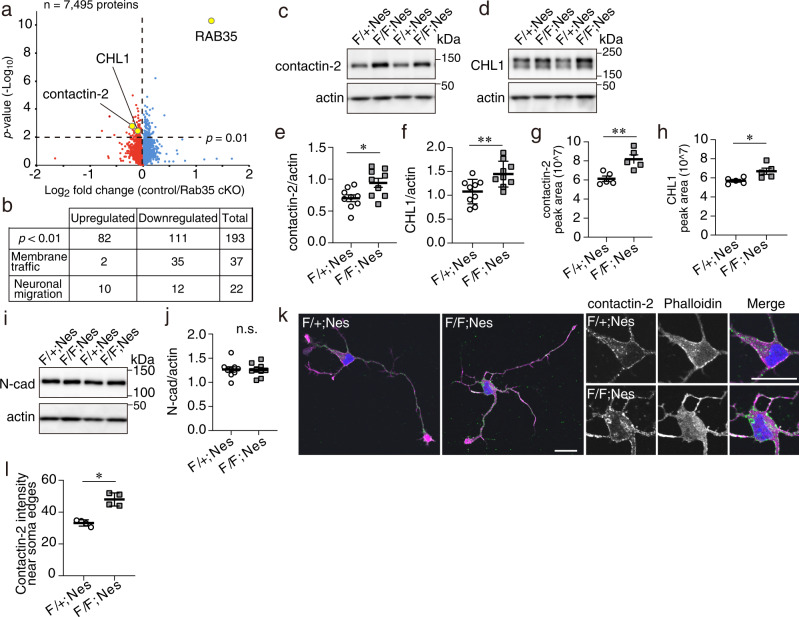
Quantitative proteomics in *Rab35* cKO P0 hippocampus. **a** Volcano plot of the TMT-based quantitative proteomes identifying the dysregulated proteins in *Rab35* cKO hippocampus in comparison with the control hippocampus (*n* = 5 mice per genotype). **b** Number of proteins identified as significantly dysregulated and as either membrane traffic-related or neuronal migration-related. **c**, **d** Western blot analysis of control and *Rab35* cKO P0 hippocampi using anti-contactin-2, anti-CHL1, and anti-actin antibodies. **e**, **f** Quantification of contactin-2 (**c**) and CHL1 (**d**) protein levels in control and *Rab35* cKO P0 hippocampi. Band intensities of the indicated proteins were normalized to those of actin (*n* = 9 mice per genotype). Unpaired Student’s *t*-test; **e**, *p* = 0.0153; **f**, *p* = 0.0095. **g**, **h** Levels of contactin-2 (**g**) and CHL1 (**h**) were quantified by targeted MS using the PRM method (*n* = 5 mice per genotype). Unpaired Student’s *t*-test; **g** *p* = 0.0053; **h** *p* = 0.0229. **i** Western blot analysis of control and *Rab35* cKO P0 hippocampus using anti-N-cadherin and anti-actin antibodies. **j** Quantification of N-cadherin protein levels in the control and *Rab35* cKO P0 hippocampus (*n* = 9 mice per genotypes). Unpaired Student’s *t*-test, *p* = 0.9020. **k** Representative images of DIV 2 hippocampal primary neurons stained for contactin-2 (green), rhodamine-phalloidin (magenta) and DAPI (blue). Scale bar, 20 μm. **l** Quantification of contactin-2 intensity at the somatic plasma membrane in control (*n* = 4) and Rab35-deficient (*n* = 4) cells. Thirty neurons from four different cultures per genotype were analyzed. Mann–Whitney *U*-test, *p* = 0.0286. Data represent the mean ± SEM; n.s. not significant (*p* > 0.05); *p < 0.05; **p < 0.01.

To gain insight into the molecular mechanism by which RAB35 contributes to neuronal migration, we assessed the differential protein expression in the hippocampus from P0 in control and *Rab35* cKO mice using a multiplexed isobaric tandem mass tag (TMT) labeling and LC-MS/MS analysis. In the P0 hippocampus, neurogenesis passed the peak, and most pyramidal neurons migrated toward their position. Among the 7495 proteins identified and quantified using P0 hippocampal proteomics, 193 were significantly upregulated or downregulated when the threshold was set to *p* < 0.01 (Fig. 5a and Supplementary Data 1). Consistent with the western blot analysis (Fig. 2a, b), we confirmed that the RAB35 protein was markedly decreased. This dataset included 37 proteins related to membrane traffic, especially the recycling and endocytic pathways, and 35 of 37 proteins, including RAB11B, RAB4A, Snx3, Atg9a, Vamp4, and RAB7 were decreased in the *Rab35* cKO hippocampus (Fig. 5b and Supplementary Table 1), suggesting that the loss of RAB35 might disturb the recycling and endocytic pathways.

In the dataset, we found that 25 neuronal migration-related proteins are affected by the loss of RAB35. Among these, contactin-2/transiently expressed the axonal surface glycoprotein-1 (TAG-1) and the close homolog of L1 (CHL1) was significantly more abundant in *Rab35* cKO hippocampus than in control mice (Fig. 5a and Supplementary Table 2). Contactin-2 and CHL1 are neural cell adhesion molecules (NCAMs) of the immunoglobulin superfamily (IgSF) belonging to the contactin and L1 subfamilies, respectively. Contactin-2 and CHL1 are present on the cell surface and mediate cell-cell and cell-substratum adhesions via the homophilic and/or heterophilic interactions with other cell adhesion molecules and the extracellular matrix to play a role in axon growth and in guiding migrating neurons in the developing brain. We confirmed that the protein levels of both contactin-2 and CHL1 were significantly increased in P0 *Rab35* cKO hippocampus using western blot analysis and parallel reaction monitoring (PRM)-based targeted mass spectrometry (Fig. 5c–h). The protein levels of other cell adhesion molecules, including the cadherin family N-cadherin, were unchanged in control and *Rab35* KO mice (Fig. 5i, j). In DIV 2 hippocampal neurons isolated from E18 embryos, contactin-2 was shown to be localized at the plasma membrane of the soma and neurites (Fig. 5k). Moreover, the fluorescence intensity of contactin-2 at the somatic plasma membrane of RAB35-deficient neurons was significantly higher when compared to control neurons (Fig. 5l). Exogenously expressed GFP-RAB35 tended to reduce the level of the contactin-2 signal on the somatic plasma membrane when compared with that in control cells; however, the difference was not statistically significant (Supplementary Fig. 2p). Thus, these results suggest that RAB35 ablation affects protein homeostasis in the developing hippocampus, resulting in increased contactin-2 and CHL1 levels, most likely due to a defect in internalization from the cell surface and/or recycling back to the plasma membrane.

## Discussion

RAB35 plays an important role in diverse cellular processes associated with dynamic membrane changes, such as endocytic recycling, cytokinesis, cell migration, and neurite outgrowth. However, the physiological function of mammalian RAB35 is yet to be investigated. This study, involving systemic and CNS-specific *Rab35*-knockout mice, identified RAB35 as an essential component in hippocampal development by regulating neuronal positioning properly.

Systemic *Rab35* knockout caused embryonic lethality until E11.5, indicating that RAB35 is essential for embryonic development. *Rab35* homozygous knockout embryos were obtained at E9.5, at the expected Mendelian frequency (Supplementary Fig. 1), suggesting that *Rab35*-knockout embryos were implanted but failed to develop. After implantation, gastrulation forms essential layers, including the ectoderm, mesoderm, and endoderm. Visceral endoderm cells exhibit high endocytic activity, supply maternal nutrients, and control signaling cascades for embryonic growth. Interestingly, RAB7 deficiency perturbs endocytic flow in visceral endoderm cells and causes reduced levels of intracellular amino acids, along with dysregulated cellular signaling, resulting in a lethal embryonic phenotype at E7^39,40^. One possible explanation for the essential nature of RAB35 during development is that *Rab35*-knockout embryos exhibit defects in recycling pathways in the visceral endoderm. Additional investigations are required to establish the role of RAB35 in mouse embryogenesis. Furthermore, we also generated CNS-specific *Rab35* cKO mice to examine the role of RAB35 in brain development. CNS-specific *Rab35* cKO mice had comparable brain weights to control mice and preserved the laminated structure of the cerebral cortex (Fig. 3). Although RAB35 is involved in neuritogenesis, neurons from *Rab35* cKO mice showed elongated axons in vitro and in vivo (Fig. 4). This discrepancy may arise because of differences in the cell lines/tissues used or differences between chronic and acute depletion of RAB35. Alternatively, other RAB proteins may compensate for the defects caused by the loss of RAB35 during early brain development.

In contrast, RAB35-deficient hippocampi showed a disorganized cell layer through the CA and DG regions (Fig. 4). Hippocampal lamination is formed under the strict spatiotemporal control of the neuronal production and the migration of neurons from the proliferative zones. The proliferative potential and morphology of radial glial cells were normal in *Rab35* cKO mice (Supplementary Fig. 2). These findings indicate that neither defective neuronal proliferation from radial glial cells nor radial processes used as a scaffold for migrating neurons are the primary causes for the malpositioning of mutant neurons. In the birthdate analysis, *Rab35* cKO neurons migrated but failed to migrate to precise locations. These data indicate that RAB35 is required to precisely distribute pyramidal cells in the developing hippocampus.

Although several RAB proteins associated with endocytosis (RAB5, 7, 11, 18, 23) have been reported to play important roles in the formation of the six-layered structure in the cerebral cortex by regulating distinct steps of neuronal migration^30–32^, the involvement of these RAB proteins in hippocampal lamination remains unknown. Interestingly, RAB35-deficient mice showed a superficially normal cerebral cortex structure but exhibited defective hippocampal lamination, suggesting that RAB35 contributes more predominantly to proper neuronal positioning in the hippocampus than in the cerebral cortex.

How does the loss of RAB35 widely perturb membrane trafficking in hippocampal neurons? One possible explanation is that the loss of RAB35 alters phosphoinositide (PI) homeostasis and leads to defects in endocytic trafficking. RAB35 is known to regulate PI homeostasis, especially PtdIns(4,5)P2 and PtdIns3P/ PtdIns(3,5)P2, in coordination with its effectors, ACAP2 and myotubularin phosphatases, respectively. ACAP2 is a GTPase-activating protein (GAP) for Arf6, which activates its effector PIP5K to synthesize PtdIns(4,5)P2. Activated RAB35 localizes to Arf6-positive compartments and then recruits ACAP2 to inactivate Arf6 in order to terminate PtdIns(4,5)P2 synthesis^3,41,42^. In contrast, recent studies have reported that RAB35 controls the turnover of PtdIns3P and PtdIns(3,5)P2 by binding to myotubularin-related protein 13- and MTMR2-containing lipid phosphatase complexes, which hydrolyze the 3-phosphate from PtdIns3P and PtdIns(3,5)P2 located on endosomes. Thus, the lack of RAB35 could provoke a local increase in PtdIns(4,5)P2, PtdIns3P, and PtdIns(3,5)P2, thereby perturbing the recycling and endocytic pathways. Indeed, the results of the proteomics analysis revealed that the levels of various endocytic regulators, such as Snx3 and Snx27, both of which bind to PtdIns3P and PIP5k1c, were significantly reduced in RAB35-deficient hippocampal lysates (Fig. 5 and Supplementary Table 1).

We also found that IgSF cell surface proteins, namely contactin-2 and CHL1, increased in the developing *Rab35* cKO hippocampus. It has been reported that CHL1 plays a role in neuronal migration in the cerebellum and caudal cerebral cortex^43–45^. During cerebellar development, the L1 family protein CHL1 controls neuronal migration via heterophilic trans-interactions of CHL1 with vitronectin and integrins. Furthermore, the cytoplasmic regions of CHL1 interacted with ezrin and ankyrin to participate in actin dynamics^46,47^. The increase or decrease in the level of another L1 family protein retarded the migration of cortical neurons^48,49^. Contactin-2 has also been reported to be involved in the migration of cortical interneurons and caudal medulla neurons^50,51^. Contactin-2 bound to L1 via cis-interaction and enabled L1 to recruit Ankyrin^52^. Given that RAB35 is required for the endocytosis and recycling of cell surface proteins, loss of RAB35 could disturb the recycling and targeting of these molecules to the appropriate locations for neuronal migration, thereby resulting in the disorderly distribution of pyramidal neurons and granule cells in the hippocampus. Moreover, RAB35 deficiency may impact intracellular actin dynamics. Alternatively, the loss of RAB35 may cause aberrant local accumulation of PI derivatives on the membrane compartments, thereby disturbing the endocytic trafficking of cell surface proteins.

Behavioral tests revealed that CNS-specific *Rab35* cKO mice have defects in a broad range of behaviors, such as anxiety-related behaviors and motor function, as well as spatial memory. (Fig. 2). The dorsal hippocampus is responsible for spatial learning and memory, whereas the ventral hippocampus functions as part of the anxiety circuitry. Thus, reduced spatial memory performance in *Rab35* cKO mice could be associated with disorganized hippocampal lamination. Several brain regions, including the amygdala, striatum, ventral hippocampus, and prefrontal cortex, modulate anxiety-related behaviors. Our data suggest that disrupted layer formation in the ventral hippocampus of *Rab35* cKO mice may affect anxiety-like behaviors. Since RAB35 is also expressed in other brain regions, including the striatum and prefrontal cortex, the loss of RAB35 in such regions may contribute to reduced anxiety-related behaviors in *Rab35* cKO mice. Therefore, further studies are needed to understand the physiological roles of RAB35 in brain development and function.

Recent studies have suggested associations between RAB35 and neurodegenerative diseases. Our proteomics data showed a significant decrease in Alzheimer’s disease-related factors LRP1 and presenilin in RAB35-deficient hippocampus. Further characterization of the physiological and pathological functions of RAB35 will provide a better understanding of the molecular pathology of neurodegenerative diseases.

## Methods

### Generation of *Rab35*-knockout mice

*Rab35*-knockout mice were generated as described previously^53^. *Rab35*-knockout ES clones were purchased from EUCOMM, with the SA-IRES-βgeo-polyA sequence inserted into the second intron of the *Rab35* gene. The targeted ES clones were identified using Southern blot analysis and injected into the BALB/c embryos to generate the *Rab35*^*geo*/+^ mice. The mice were back-crossed more than six generations to the C57BL/6 background. To generate the *Rab35*^*flox*/+^ and *Rab35*^−/+^ mice, the *Rab*35^*geo*/+^ mice were crossed with *Act-Flp-e* and *CAG-Cre* transgenic mice (Jackson Laboratory), respectively. To generate the CNS-specific *Rab35*-knockout mice, *Rab35*^*flox*/+^ mice were crossed with *Nestin-Cre* transgenic mice^33^. The genotypes of each mouse line were confirmed through PCR using the following primers: primer 1 (5′ arm), 5′-ACACTTCACATGGCTCTCTGGTCC-3′; primer 2 (3′ arm), 5′-CTCTAGCAGACCCACAATGCGAGC-3′; primer 3 (LAR3), 5′-CAACGGGTTCTTCTGTTAGTCC-3′; and primer 4 (40 R), 5′-TGAACTGATGGCGAGCTCAGACC-3′. The *cre* gene was identified through PCR using the following primers: forward, 5′-AGGTTCGTTCACTCATGGA-3′; and reverse, 5′-TCGACCAGTTTAGTTACCC-3′. All animal procedures were approved and performed in accordance with the guidelines of the Animal Care and Experimentation Committee of Gunma University, Japan (approval no. 19-001). Mice were bred at the Bioresource Center of Gunma University, Graduate School of Medicine, Japan.

### Antibodies

The following primary antibodies were used for immunoblotting: 1:1000 RAB35 (rabbit; Cell Signaling, 9690 S), 1:10,000 Actin [C4] (mouse; Merck-Millipore, MAB1501), 1:10,000 GAPDH [6C5] (mouse; Merck-Millipore, MAB374), 1:1000 contactin-2/TAG-1 (goat; R&D systems, AF4439), 1:1000 CHL1 (goat; R&D systems, AF2147), and 1:1000 N-cadherin [32/N-cadherin] (rabbit; BD, 610920). The following primary antibodies were used for immunostaining: 1:100 NeuN [A60] (mouse; Merck-Millipore, MAB377), 1:200 GFAP (rabbit, Frontier Institute, GFAP-Rb-Af800), 1:200 β-galactosidase (mouse, Promega, Z3781), 1:100 Cux1 (rabbit; Santa Cruz, sc13024), 1:100 Ctip2 (rat; Abcam, ab18465), 1:300 phosphorylated neurofilament [SMI-31] (mouse; Covance, SMI-31R), 1:500 Tbr2 (rabbit; Abcam, ab23345), 1:300 Pax6 (rabbit; BioLegend, PRB-278P), 1:200 phospho-Histone H3 (Ser10) [RR002] (rabbit; Cell Signaling, 9701), and 1:100 Nestin [Rat-401] (mouse; Invitrogen, 14-5843-82). The following secondary antibodies were used for immunostaining: goat anti-rat Alexa Fluor-488, donkey anti-mouse Alexa Fluor-488, donkey anti-rabbit Alexa Fluor-488, donkey anti-mouse Alexa Fluor-594, and donkey anti-rabbit Alexa Fluor-594 (all antibodies are from Life Technologies).

### X-Gal staining

Fresh brains were fixed with 2% paraformaldehyde (PFA)/phosphate-buffered saline (PBS) for 30 min–2 h at 4 °C and transferred sequentially to a series of 5, 10, and 30% sucrose in PBS overnight at 4 °C. The brains were embedded in Tissue-Tek OCT compound (Sakura Finetek) and cryosectioned at 30-μm thickness. The sections were mounted on glass slides and washed with solution C (0.01% sodium deoxycholate, 0.02% NP-40, 5 mM EGTA, and 2 mM MgCl_2_ in PBS) and incubated in solution D (0.5 mg/ml X-gal, 10 mM K_3_[Fe(CN)_6_], and 10 mM K_4_[Fe(CN)_6_] in solution C) overnight at 37 °C. After fixation with 4% PFA/PBS for 10 min, the sections were subjected to nuclear-fast red staining. Images were acquired using a BX50 microscope (Olympus).

### Immunohistochemical analysis

Adult mice were transcardially perfused with 4% PFA/PBS, and the brains were postfixed overnight at 4 °C. For paraffin sections, the fixed brains were dehydrated in 70% ethanol, embedded in paraffin, and sectioned at 4-μm thickness. The sections were subjected to antigen retrieval using an autoclave in 0.01 M citrate buffer at 105 °C for 15 min and incubated with 3% H_2_O_2_/MeOH for 30 min. After blocking with 5% BSA/PBS for 30 min, the sections were incubated with anti-NeuN [A60] antibody in 1% BSA/PBS at 4 °C overnight, followed by incubation with EnVision Plus System-HRP Labeled Polymer Anti-Mouse kit (DAKO Cytomation, K4000) for 1 h. The signal was visualized using the Liquid DAB substrate chromogen system (DAKO Cytomation). After rinsing with water, the sections were counterstained with hematoxylin. Images were acquired using a BZ-9000 microscope (Keyence) or a BX50 (Olympus). For quantification of the cortical thickness and the number of NeuN-positive cells, images of 300-μm width cerebral cortex were analyzed using ImageJ software (National Institutes of Health). For bin analysis of the hippocampus, images of 200-μm width × 500-μm length in CA1 and CA3 or 200-μm width × 250-μm length in DG were divided into five equal bins; then, NeuN-positive cell numbers in each bin were counted using ImageJ software (National Institutes of Health). For frozen sections, the brains isolated from E15 embryos and P0 and P6 pups were fixed in 4% PFA/PBS at 4 °C overnight, dehydrated in 30% Sucrose/PBS at 4 °C overnight, embedded in Tissue-Tek OCT compound, and cryosectioned. The 10-μm thick sections were subjected to antigen retrieval in 0.01 M citrate buffer (pH 6.0) at 90 °C for 10 min (for Cux1, Ctip2, Pax6, and Tbr2 staining) or in Histo VT one (NACALAI TESQUE) at 70 °C 10 min (for pHH3 staining), followed by permeabilization with 0.1% Triton X/PBS for 10 min (Pax6 and Tbr2 staining). After blocking with 1% BSA/PBS for 10 min, the sections were incubated with primary antibodies at 4 °C overnight. After washing with PBS, the sections were incubated with 1% BSA/PBS for 10 min, followed by incubation with secondary antibodies and DAPI. The images were acquired using a BZ-9000 microscope (Keyence) or a BX50 (Olympus). Pax6-, Tbr2-, or pHH3-positive cells in 250-μm width CA or 100-μm width DG were analyzed using ImageJ software. Quantitative analyses were conducted blind to genotype. Basically, two sections per animal were analyzed. For β-galactosidase staining, 20-μm thick sagittal sections were rinsed with PBS, permeabilized, and blocked with 0.1% Triton X-100/5% NDS/PBS for 20 min. the sections were incubated with anti-β-galactosidase and anti-NeuN or GFAP antibodies in 0.1% Triton X-100/1% NDS/PBS at 4 °C overnight. After washing with PBS, the sections were incubated with secondary antibodies and DAPI for 1 h. the images were acquired using FV1200 (Olympus).

### Measurement of the thickness of the VHC

Coronal sections (4-μm thick) at Bregma −0.35 mm placed on slides were sequentially soaked in xylene (three times for 5 min), 100% ethanol (three times for 3 min), and 95% ethanol (5 min). The sections were then incubated with 0.1% Luxol Fast Blue/95% ethanol at 50 °C overnight. After washing with water, the sections were soaked in 0.05% lithium carbonate for 5 s, dehydrated as above, and counterstained with 0.1% crystal violet (Sigma, C5042). Images were acquired using a BZ-9000 microscope (Keyence) and analyzed using the ImageJ software.

### Fluorescent Nissl staining

Briefly, OCT-embedded brains were sectioned in the coronal plane at 50-μm thickness, and slices were transferred to 24-well plates in PBS. The slices were extensively rinsed with PBS and permeabilized with 0.1% Triton X-100/PBS for 20 min. After washing with PBS, the sections were incubated in Neurotrace 530/615 (1:200; Thermo Fisher Scientific; N21482) for 60 min and rinsed with PBS. The images were acquired using a BZ-9000 (Keyence) microscope.

### Immunoblot analysis

Isolated mouse brains were homogenized in cold homogenate buffer (50 mM Tris-HCl pH 8.0, 0.32 M sucrose, 1 mM EDTA, and 1 mM phenylmethylsulfonyl fluoride with protease inhibitor cocktail) using a Dounce homogenizer. After the addition of an equal amount of lysis buffer (50 mM Tris-HCl pH 7.5, 250 mM NaCl, 1 mM EDTA, 1% Triton X-100, 0.5% sodium deoxycholate, 0.1% SDS, and 1 mM phenylmethylsulfonyl fluoride with protease inhibitor cocktail), the lysates were incubated on ice for 30 min and centrifuged at 15,000 rpm for 15 min at 4 °C. The supernatants were mixed with the sample buffer, incubated for 30 min at 37 °C, subjected to SDS-PAGE, and blotted onto polyvinylidene fluoride (PVDF) membranes. After blocking with 5% skim milk/TBST (50 mM Tris-HCl pH 8.0, 150 mM NaCl, and 0.05% Tween-20), the membranes were incubated with primary and secondary antibodies in 1% skim milk/TBST. The intensities of the immunoreactive bands were quantified by ImageQuant TL (GE Healthcare).

### Hippocampal primary neuron culture

Hippocampi were dissected from E18.5 mouse embryos, treated with 0.15% trypsin (Gibco) and 0.1% Collagenase (Wako) at 30 °C for 30 min, and then triturated gently. In order to measure the lengths of the axons and neurites, 2 × 10^4^ cells (for neurite lengths) or 6 × 10^4^ cells (for contactin-2 intensity) were plated on poly-l-lysine-coated 3.5-cm dishes (for neurite lengths) or coverslips in 3.5-cm dishes (for contactin-2 intensity) and cultured in a neurobasal medium (Gibco, 21103049) supplemented with 2% B27 (Gibco, 17504001) and 0.5 mM glutamine (Sigma-Aldrich) for DIV 3 or DIV 2. To measure neurite length, cells were fixed with 4% PFA/PBS for 30 min, permeabilized with 0.1% Triton-100/PBS for 15 min, blocked with 5% NDS/PBS for 1 h, and incubated with an anti-phosphorylated neurofilament [SMI-31] antibody and rhodamine-conjugated phalloidin (Thermo Fisher Scientific, R415) overnight at 4 °C. The cells were then incubated with a secondary antibody and DAPI for 1 h. Images were acquired using an FV1000 confocal microscope (Olympus). Axon length (phosphorylated neurofilament-positive neurites) and total neurite length (rhodamine-conjugated phalloidin-positive neurites) were measured using the ImageJ software. For contactin-2 staining, cells were fixed with 4% PFA/PBS for 15 min, permeabilized with 0.05% saponin/PBS for 15 min, blocked with 5% NGS/PBS for 1 h, and incubated with an anti-contactin-2 antibody and rhodamine-conjugated phalloidin overnight at 4 °C. Subsequently, the cells were incubated with a secondary antibody and DAPI for 1 h. Images were acquired using an FV1200 confocal microscope (Olympus). To express exogenous GFP and GFP-RAB35, 8 × 10^4^ cells were transfected with pcDNA3.1 GFP or GFP-RAB35 plasmids at DIV 1 using HilyMax (DOJINDO) according to the manufacturer’s instructions and fixed at DIV 3. Quantification of contactin-2 intensity at the somatic plasma membrane was performed using the ImageJ software.

### Behavioral analysis

Behavioral tests were performed with the control (*Rab35*^*flox/+*^; *Nestin-Cre*) and *Rab35* cKO (*Rab35*^*flox/flox*^; *Nestin-Cre*) male littermates at 12–18 weeks of age during the light period of the 12-h light/dark cycle. Mice were handled 5 min per day for 4–5 days prior to a battery of behavioral tests and habituated to the testing room for at least 30 min before each test. Behavioral tests were carried out by an experimenter blinded to the genotype. In the open field test, each mouse was placed in one corner of the open field apparatus, consisting of white PVC (50 × 50 × 40 cm, width × depth × height; O’Hara & Co.) and illuminated at 70 lux, and was allowed to move freely for 15 min. Data were recorded and analyzed using Time OFCR1 software (O’Hara & Co.). The elevated plus maze apparatus (O’Hara & Co.) consisted of two open arms and two closed arms (25 × 5.5 cm each) with 15 cm high transparent walls. The maze was placed 50 cm above the floor and illuminated at 70 lux. Each mouse was placed in the center of the maze facing an open arm and was allowed to move freely in the maze for 10 min. Data were recorded and analyzed using ImageJ EP1 software (O’Hara & Co.). In the rotarod test, mice were placed on a rod (O’Hara & Co.) rotating at 4 rpm. The speed of rotation was accelerated from 4 to 40 rpm over a 300 s period. Mice were trained in three trials per day with a 1.5 h interval between trials for two sequential days. The time at which a mouse fell from the rod was measured. The elevated plus maze apparatus (O’Hara & Co.) consisted of two open arms and two closed arms (25 × 5.5 cm each) with 15 cm high transparent walls. The maze was placed 50 cm above the floor and illuminated at 70 lx. Each mouse was placed in the center of the maze facing an open arm and allowed to move freely for 10 min. Data were recorded and analyzed using the ImageJ EP1 software (O’Hara & Co.). The Y maze apparatus was composed of three opaque arms and illuminated at 70 lx. Each mouse was placed at the end of one arm and allowed to move freely during an 8 min session. The percentage of spontaneous alternations was calculated as follows:

1 $${{{{{\rm{Spontaneous}}}}}}\,{{{{{\rm{alternation}}}}}}\, \% =\frac{{{{{{\rm{the}}}}}}\,{{{{{\rm{number}}}}}}\,{{{{{\rm{of}}}}}}\,{{{{{\rm{spontaneous}}}}}}\,{{{{{\rm{alternation}}}}}}}{{{{{{\rm{total}}}}}}\,{{{{{\rm{number}}}}}}\;{{{{{\rm{of}}}}}}\; {{{{{\rm{arm}}}}}}\; {{{{{\rm{entries}}}}}}-2}\times 100$$

Data were recorded and analyzed using the Time YM1 software (O’Hara & Co.). The Barnes maze apparatus (O’Hara & Co.) is a circular platform (1 m in diameter) with 16 equally spaced holes along the perimeter, elevated 75 cm above the floor. A black escape box was located under one of the holes, which represented the target. The target hole (escape hole) was consistent for each mouse but randomized between mice. Illumination was maintained at 200 lx. Four spatial cues with distinct colors and shapes were hung from the walls of the testing room during the training phase and probe test but not during the habituation phase. During habituation (day 1), each mouse was placed in an opaque cylindrical start chamber at the center of the maze. After 10 s, the mouse was allowed to freely explore the maze for 3 min and then gently guided to the escape box. During the acquisition trials (days 2–5), 10 s after being placed in the start chamber, each mouse was allowed to freely explore the maze for 3 min per trial. Each mouse was given three trials with a 15 min interval between trials for four sequential days. Trials ended when the mouse entered the target hole or when the 3 min had elapsed. Mice that did not enter the escape box were gently guided to the target hole and spent 1 min. During the probe test (day 6), each mouse freely explored the maze without an escape box for 3 min. Data were recorded and analyzed using the Time BCM software (O’Hara & Co.). The probe test used 90 s of 3 min data for analysis.

### EdU labeling birthdate analysis

Pregnant mice at E14.5 were intraperitoneally injected with 50 mg/kg body weight of 5-ethynyl-2′-deoxyuridine (EdU; Thermo Fisher Scientific, A10044). The brains of the P12 littermates were isolated and fixed in 4% PFA/PBS at 4 °C overnight, dehydrated in 30% sucrose/PBS at 4 °C overnight, embedded in the OCT compound, and cryosectioned at 20-μm thickness. EdU detection was performed using a Click-it EdU Alexa 594 imaging kit protocol (Thermo Fisher Scientific, C10339) according to the manufacturer’s instructions. Images were acquired by using a BZ-9000 microscope (Keyence). For the quantification, the hippocampus (400-μm width) and cortex (500-μm width) were divided into five or ten equal bins and quantified the number of EdU-positive cells. Basically, two sections per animal were analyzed using ImageJ software.

### Hippocampal proteomics

The hippocampi from the control (*Rab35*^*flox/+*^; Nestin-Cre) and *Rab35* cKO mice groups at P0 (*n* = 5 biological replicates per genotype) were lysed in a buffer containing 6 M guanidine hydrochloride, 100 mM HEPES-NaOH (pH 8.0), 10 mM TCEP, and 40 mM chloroacetamide. The lysates were dissolved through heating and sonication, followed by centrifugation at 15,000 rpm for 15 min at 4 °C. Proteins (100 µg each) were purified using methanol/chloroform precipitation and solubilized through the addition of 20 µL of 0.1% RapiGest SF (Waters) in 50 mM triethylammonium bicarbonate. After repeated sonication and vortexing, the proteins were digested with 1 µg of trypsin/Lys-C mix (Promega) for 16 h at 37 °C. The peptide concentrations were determined using the Pierce quantitative colorimetric peptide assay (Thermo Fisher Scientific). Approximately 25 µg of peptides for each sample was labeled with 0.2 mg of TMT10-plex reagents (Thermo Fisher Scientific) for 1 h at 25 °C. After the reaction was quenched with hydroxylamine, all the TMT-labeled samples were pooled, acidified with trifluoroacetic acid (TFA), and fractionated using the Pierce high pH reversed-phase peptide fractionation kit (Thermo Fisher Scientific) according to the manufacturer’s instructions. Nine fractions were collected using 5, 10, 12.5, 15, 17.5, 20, 22.5, 25, and 80% acetonitrile (ACN). Each fraction was evaporated in a SpeedVac concentrator and dissolved in 0.1% TFA and 3% ACN.

LC-MS/MS analysis of the resultant peptides (1 µg each) was performed on an EASY-nLC 1200 UHPLC connected to a Q Exactive Plus mass spectrometer through a nanoelectrospray ion source (Thermo Fisher Scientific). The peptides were separated with a linear gradient from 4–20% ACN for min 0–180 and 20–32% ACN for min 180–220, followed by an increase to 80% ACN during min 220–230. The mass spectrometer was operated in a data-dependent acquisition mode with a top 15 MS/MS method. MS1 spectra were measured with a resolution of 70,000, an automatic gain control (AGC) target of 3 × 10^6^, and a mass range of 375–1400 *m/z*. HCD MS/MS spectra were acquired at a resolution of 35,000, an AGC target of 1 × 10^5^, an isolation window of 0.4 *m/z*, a maximum injection time of 100 ms, and a normalized collision energy of 34. Dynamic exclusion was set to 30 s. Raw data were directly analyzed against the SwissProt database restricted to *M. musculus* using Proteome Discoverer version 2.3 (Thermo Fisher Scientific) with Mascot search engine version 2.5 (Matrix Science) for identification and TMT quantification. The search parameters were as follows: (a) trypsin as an enzyme with up to two missed cleavages; (b) precursor mass tolerance of 10 ppm; (c) fragment mass tolerance of 0.02 Da; (d) TMT of lysine and peptide N-terminus and carbamidomethylation of cysteine as fixed modifications; and (e) oxidation of methionine as a variable modification. Peptides were filtered at a false-discovery rate (FDR) of 1% using the percolator node.

To quantify contactin-2 and CHL1, at least three peptides/proteins were measured using PRM, an MS/MS-based targeted quantification method using high-resolution MS. Targeted MS/MS scans were acquired by a time-scheduled inclusion list at a resolution of 70,000, an AGC target of 2 × 10^5^, an isolation window of 4.0 *m/z*, a maximum injection time of 500 ms, and a normalized collision energy of 27. Time alignment and relative quantification of the transitions were performed using PinPoint version 1.4 (Thermo Fisher Scientific).

### Statistics and reproducibility

Statistical analyses were performed using GraphPad Prism 8 (GraphPad Software) and EZR software^54^. Sample sizes were determined based on previous studies using similar experiments. The experimenters were blinded during the acquisition of imaging data, quantification, and behavior tests. Data, including outliners, were checked for normality using the Shapiro–Wilk test and the equality of variance was checked using *F*-test. The means between the two groups were compared using a parametric test (two-tailed unpaired Student’s *t*-test or Welch’s *t*-test) or a nonparametric test (two-tailed Mann–Whitney *U*-test). The means between the three groups were compared using a one-way ANOVA. Data from multiple groups were analyzed using two-way repeated-measures ANOVA with Bonferroni multiple comparison tests. In the dot plots, the bars represent mean ± SEM. In the box and whisker plots, the boxes represent the interquartile range (25th to 75th percentile) and the bars in the boxes represent the median. The whiskers cover the range from minimum to maximum. The dots in the graphs show individual data points. Individual information regarding the sample size and statistical tests is described in the figure legends. Statistical significance was set as *p* < 0.05. All experiments except for the western blot experiment of E13.5 brains and the proteomic analysis were performed at least two times with similar results.

### Reporting summary

Further information on research design is available in the Nature Portfolio Reporting Summary linked to this article.

## Supplementary information

Supplementary information

Description of Additional Supplementary Files

Supplementary Data 1

Supplementary Data 2

Reporting Summary

## Data Availability

The data shown in these findings are available from the authors by request. The proteomics data in this study has been deposited in jPOST. The accession numbers are PXD041290 for ProteomeXchange and JPST002112 for jPOST. The original uncropped blots and PCR images can be found in Supplementary Figs. 3,  4. Source data for the graphs can be found in Supplementary Data 2.
