# Supplementary material for: RAB35 is required for murine hippocampal development and functions by regulating neuronal cell distribution
Source: Commun Biol. 2023 Apr 21;6:440. doi: 10.1038/s42003-023-04826-x (PMC10121692; doi:10.1038/s42003-023-04826-x)
Supplement: Supplementary file 1 — Supplementary information [file 42003_2023_4826_MOESM1_ESM.docx]

**Supplementary information**


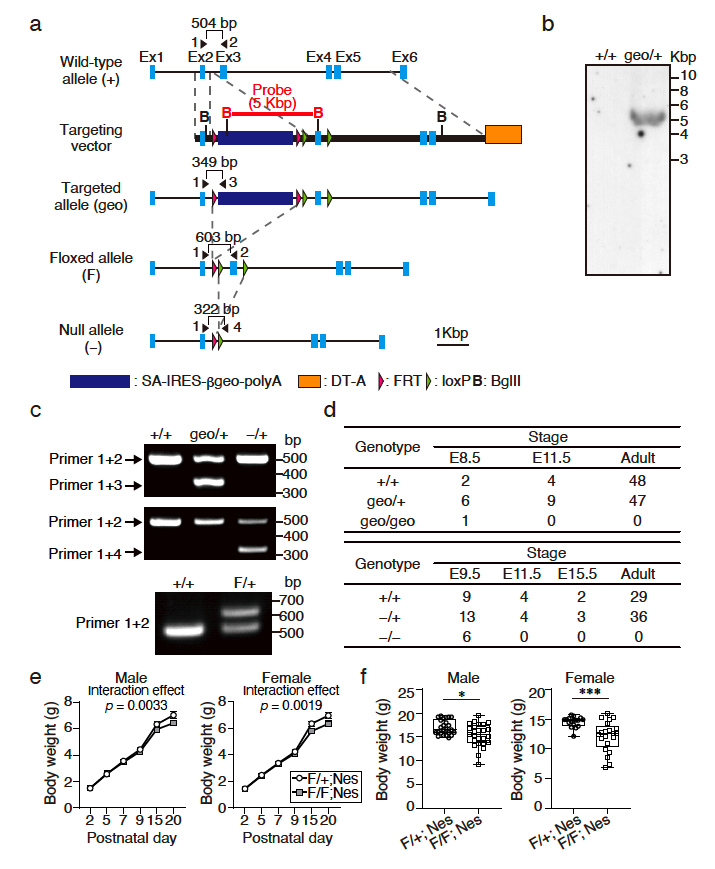


**Supplementary Figure 1. RAB35 is essential for early mouse development.**

**a** Schematic diagram of *Rab35* targeting strategies. Restriction maps of the wild-type allele, the targeting vector, and the targeted allele. The indicated probe containing neomycin fragment hybridizes with a 5 kb BglII fragment from the targeted allele. Arrowheads indicate the position of PCR primers to identify the genotypes. **b** Southern blot analysis of the targeted ES clones. Genomic DNA from *Rab35*^+/+^ and *Rab35^geo^*^/+^ clones was digested with BglII for hybridization with the probe shown in **a**. **c** PCR using genomic DNA from the tails of control and knockout mice. Primer sets for PCR are shown as arrowheads in **a**. **d** Genotypic distribution analysis of the progeny from *Rab35^geo^*^/+^ or *Rab35*^−/+^ intercrosses. **e** Body weights of control (male, *n* = 14; female, *n* = 14) and *Rab35* cKO mice (male, *n* = 14; female, *n* = 26) from P2 to P20. Two-way repeated-measures ANOVA; interaction effect, F(5, 130) = 3.760 and *p* = 0.0033 (male); interaction effect, F(5, 190) =3.981 and *p* = 0.0019 (female). **f** Body weights of control (male, *n* = 24 male; female, *n* = 17) and Rab35 cKO (male, *n* = 26; female, *n* = 21) mice at 8 weeks. Mann-Whitney test; male, *p* = 0.014; female, *p* = 0.0009. Data represent mean ± SEM or box and whisker plots; box plot shows 25^th^ and 75^th^ percentiles (boxes), median (horizontal bar), and minimum to maximum (whiskers); n.s., not significant (*p* > 0.05); **p* < 0.05; ****p* < 0.001.


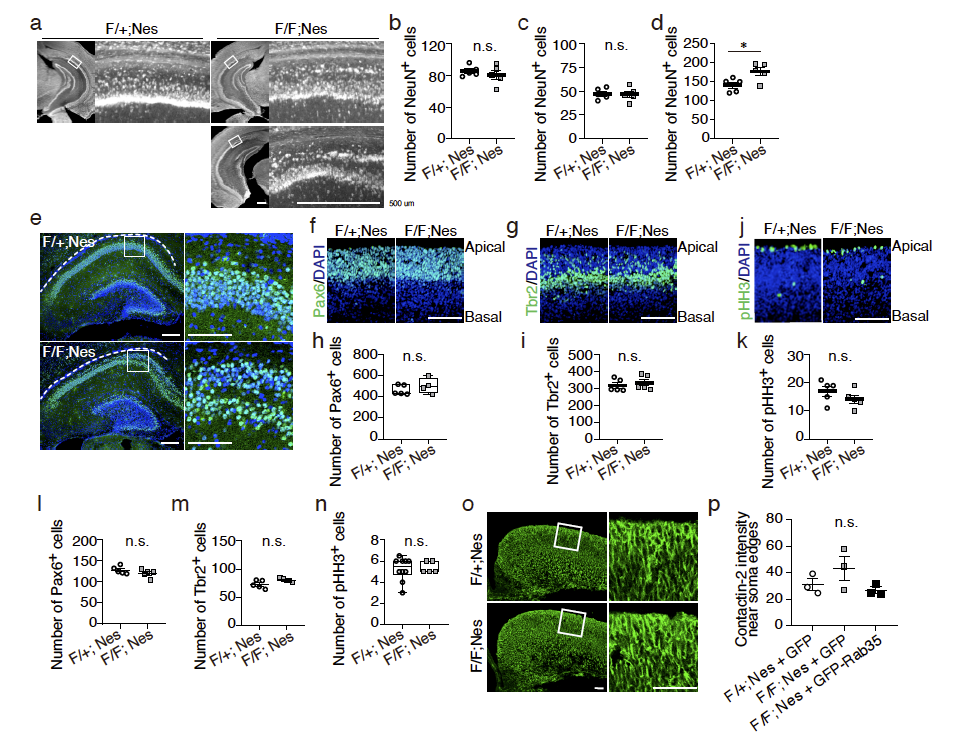


**Supplementary Figure 2. The cell proliferation ability is maintained in *Rab35* cKO hippocampi.**

**a** Representative images of the ventral hippocampi of 2-month-old control and *Rab35* cKO mice stained with NeuroTrace. Scale bar, 500 μm. **b**–**d** Number of NeuN-positive cells in 200-μm wide in hippocampal CA1 (**b**), CA3 (**c**), and DG (**d**) of control (*n* = 5) and *Rab35* cKO (*n* = 5) mice. Unpaired Student’s *t* test; **b**, *p* = 0.4597; **c**, *p* = 0.9095; **d**, *p* = 0.0293. **e** Representative images of P6 hippocampi of control and *Rab35* cKO mice stained with Ctip2 (green) and DAPI (blue). Scale bar, 400 μm. **f**, **g** Representative images of sagittal sections of E15.5 hippocampal CA regions stained for Pax6 (**f**, green), Tbr2 (**g**, green), and DAPI (blue). Scale bar, 200 μm. **h**, **i** Number of Pax6-positive cells (**h**) or Tbr2-positive cells (**i**) per 250 μm width in control (*n* = 5) and *Rab35* cKO (*n* = 4) CA regions. Mann-Whitney U test, *p* = 0.9048; unpaired Student’s *t* test, *p* = 0.5392. **j** Representative images of sagittal sections of E15.5 hippocampi CA regions stained for pHH3 (green) and DAPI (blue). Scale bar, 200 μm. **k** Number of pHH3-positive cells per 250 μm in the control (*n* = 5) and Rab35 cKO (*n* = 5) CA regions. Unpaired Student’s *t* test; *p* = 0.2602. **l**–**n** Number of Pax6-positive cells (**l**), Tbr2-positive cells (**m**) and pHH3-positive cells in DG (**n**) per 100 μm width in control (*n* = 5) and Rab35 cKO (*n* = 4) mice. Unpaired Student’s *t* test; **l**, *p* = 0.2246; **m**, *p* = 0.0683. Mann-Whitney U test; **n**, *p* > 0.99. **o** Representative images of the sagittal sections of E15.5 hippocampi stained for Nestin (green). Scale bar, 50 μm. **p** Quantification of contactin-2 intensity at the somatic plasma membrane in control (*n* = 3) and *Rab35*-deficient (*n* = 3) cells expressing GFP or GFP-RAB35. Five Fifteen neurons from three different cultures were measured per group. Mann-Whitney U test, *p* = 0.0286. Data represent mean ± SEM or box and whisker plots; n.s., not significant (*p* > 0.05); **p* < 0.05.

**
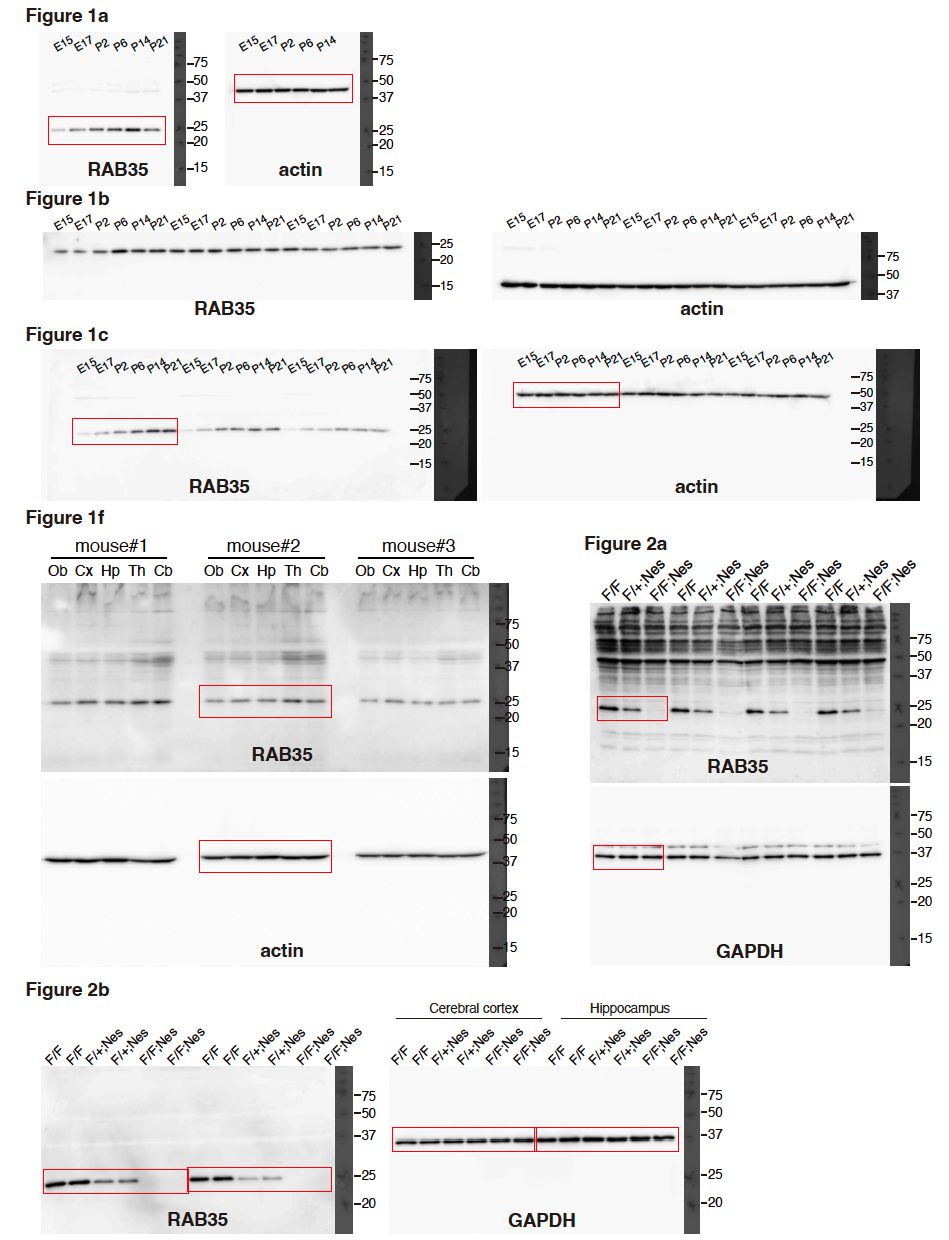
**

**Supplementary Figure 3. Uncropped images related to Fig. 1 and Fig. 2.**

The red rectangles represent the lanes shown in the figures.

**
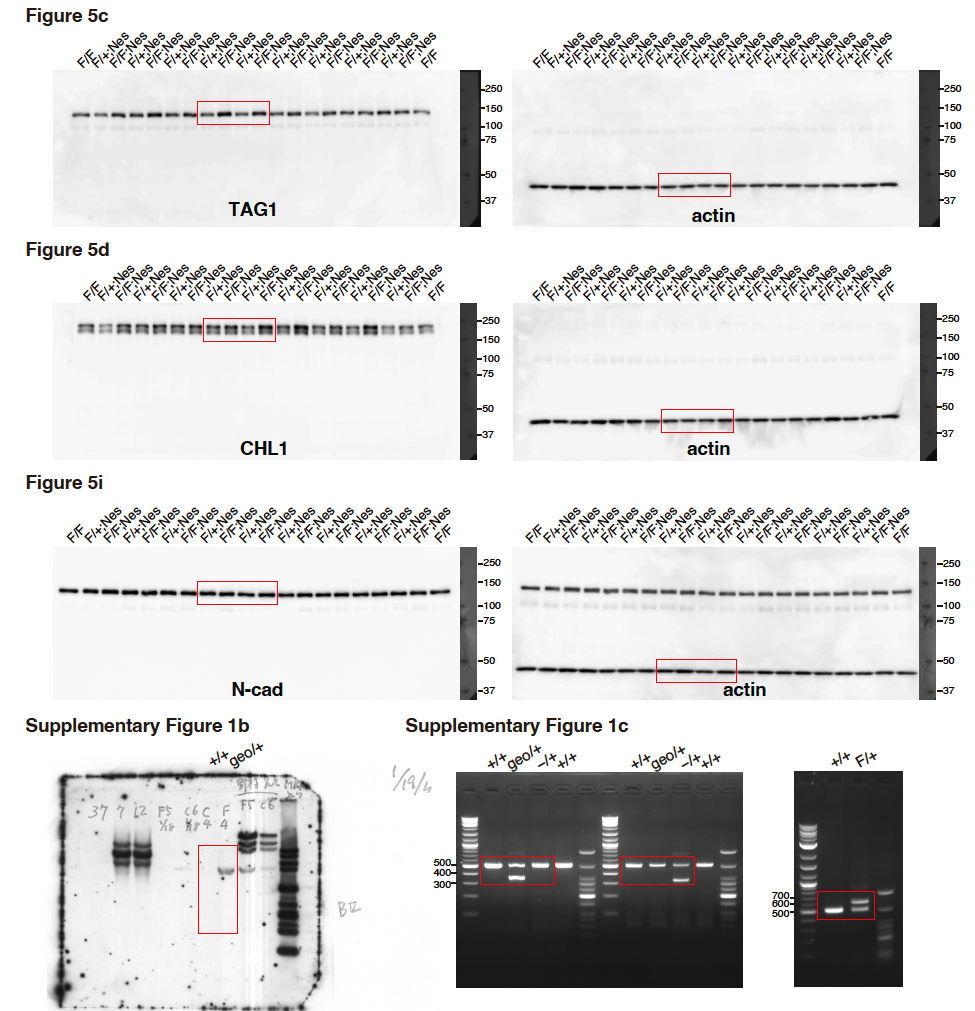
**

**Supplementary Figure 4. Uncropped images related to Fig. 5 and Supplementary Fig. 1.**

The red rectangles represent the lanes shown in the figures.

| Rab35 cKO vs. Ctrl | | |
| --- | --- | --- |
| Upregulated | Downregulated | |
| Fn1 | Rab35 | Stx12 |
| Ralbp1 | Snx3 | Rab1b |
|  | Sort1 | Vti1b |
|  | Atg9a | Snx6 |
|  | Rab4a | Vapa |
|  | Rab11b | Trappc2l |
|  | Vamp4 | Stx16 |
|  | Sorcs2 | Vps45 |
|  | Scamp1 | Pip5k1c |
|  | Rab1a | Rhoa |
|  | Vapb | Rab2b |
|  | Cav1 | Snx2 |
|  | Rab23 | Myo1c |
|  | Rab7 | Appl1 |
|  | Scamp5 | Vps36 |
|  | Rab31 | Ap2a1 |
|  | Snx27 | Ralb |
|  | Stx1 |  |

**Supplementary Table 1. Membrane traffic-related proteins dysregulated in the *Rab35* cKO P0 hippocampus.**

| Rab35 cKO vs. Ctrl | |
| --- | --- |
| Upregulated | Downregulated |
| Sparcl1 | Pdgfra |
| Cntn2 | Sort1 |
| Nes | Rab11b |
| Mdk | Cav1 |
| Sema3c | Rab23 |
| Chl1 | Rab7 |
| Fn1 | Alcam |
| Gpr56; Adgrg1 | Rap1b |
| Ptn | Rheb |
| Myh10 | Rhoa |
| Agrn | Ap2a1 |
|  | Ralb |
|  | Ephb2 |

**Supplementary Table 2. Neuronal migration-related proteins dysregulated in the *Rab35* cKO P0 hippocampus.**
