# Supplementary material for: RAB35 is required for murine hippocampal development and functions by regulating neuronal cell distribution
Source: Commun Biol. 2023 Apr 21;6:440. doi: 10.1038/s42003-023-04826-x (PMC10121692; doi:10.1038/s42003-023-04826-x)
Supplement: Supplementary file 2 — Description of Additional Supplementary Files [file 42003_2023_4826_MOESM2_ESM.pdf]

Description of Additional Supplementary Files

File name: Supplementary Data 1

Description: Proteins dysregulated in the Rab35 cKO P0 hippocampus.

File name: Supplementary Data 2

Description: Source data for graphs presented in the paper.
