# Supplementary material for: RAB35 is required for murine hippocampal development and functions by regulating neuronal cell distribution
Source: Commun Biol. 2023 Apr 21;6:440. doi: 10.1038/s42003-023-04826-x (PMC10121692; doi:10.1038/s42003-023-04826-x)
Supplement: Supplementary file 5 — Reporting Summary [file 42003_2023_4826_MOESM5_ESM.pdf]

nature portfolio

Corresponding author(s): Ken Sato

Last updated by author(s): Apr 3, 2023

Reporting Summary

Nature Portfolio wishes to improve the reproducibility of the work that we publish. This form provides structure for consistency and transparency in reporting. For further information on Nature Portfolio policies, see our Editorial Policies and the Editorial Policy Checklist.

Statistics

For all statistical analyses, confirm that the following items are present in the figure legend, table legend, main text, or Methods section.

n/a	Confirmed	
☐	☒	The exact sample size (*n*) for each experimental group/condition, given as a discrete number and unit of measurement
☐	☒	A statement on whether measurements were taken from distinct samples or whether the same sample was measured repeatedly
☐	☒	The statistical test(s) used AND whether they are one- or two-sided<br>*Only common tests should be described solely by name; describe more complex techniques in the Methods section.*
☒	☐	A description of all covariates tested
☐	☒	A description of any assumptions or corrections, such as tests of normality and adjustment for multiple comparisons
☐	☒	A full description of the statistical parameters including central tendency (e.g. means) or other basic estimates (e.g. regression coefficient) AND variation (e.g. standard deviation) or associated estimates of uncertainty (e.g. confidence intervals)
☐	☒	For null hypothesis testing, the test statistic (e.g. *F*, *t*, *r*) with confidence intervals, effect sizes, degrees of freedom and *P* value noted<br>*Give P values as exact values whenever suitable.*
☒	☐	For Bayesian analysis, information on the choice of priors and Markov chain Monte Carlo settings
☒	☐	For hierarchical and complex designs, identification of the appropriate level for tests and full reporting of outcomes
☒	☐	Estimates of effect sizes (e.g. Cohen's *d*, Pearson's *r*), indicating how they were calculated

Our web collection on statistics for biologists contains articles on many of the points above.

Software and code

Policy information about availability of computer code

Data collection	ImageQuant LAS4000 mini (v1.2) was used for acquisition of the western blot images. FV10-ASW (v4.2), OLYMPUS cellSens Dimension (v1.18) and Keyence BZ-II Viewer (v1.42) were used for acquisition of the images data.
Data analysis	ImageQuant TL was used for quantification of band intensities from western blot images. ImageJ (v1.51-1.53) was used for our image data analysis. GraphPad PRISM (v8.0c) and EZR (v1.30) were used for statistical analysis. Proteome Discoverer (v2.3) with Mascot search engine (v2.5) were used for the identification and TMT quantification. PinPoint (v1.4) were used for PRM analysis.

For manuscripts utilizing custom algorithms or software that are central to the research but not yet described in published literature, software must be made available to editors and reviewers. We strongly encourage code deposition in a community repository (e.g. GitHub). See the Nature Portfolio guidelines for submitting code & software for further information.

Data

Policy information about availability of data

All manuscripts must include a data availability statement. This statement should provide the following information, where applicable:

- Accession codes, unique identifiers, or web links for publicly available datasets
- A description of any restrictions on data availability
- For clinical datasets or third party data, please ensure that the statement adheres to our policy

The data analyzed in this study are available from the corresponding author. The proteomics data in this study can be available in jPOST.

March 2021

Human research participants

Policy information about studies involving human research participants and Sex and Gender in Research.

Reporting on sex and gender	No human research participants were involved in this study.
Population characteristics	No human research participants were involved in this study.
Recruitment	No human research participants were involved in this study.
Ethics oversight	No human research participants were involved in this study.

Note that full information on the approval of the study protocol must also be provided in the manuscript.

Field-specific reporting

Please select the one below that is the best fit for your research. If you are not sure, read the appropriate sections before making your selection.

☒ Life sciences ☐ Behavioural & social sciences ☐ Ecological, evolutionary & environmental sciences

For a reference copy of the document with all sections, see nature.com/documents/nr-reporting-summary-flat.pdf

Life sciences study design

All studies must disclose on these points even when the disclosure is negative.

Sample size	No statistical methods were used to pre-determine sample sizes for our experiments. Our sample sizes were determined based on a previous study using similar experiments.
Data exclusions	No data were excluded from any analysis.
Replication	We performed at least two independent trials with different litter mates, and/or examined with < n = 3 biological replicates. Exceptions were the western blot analysis of E13.5 embryonic brains and the mass spectrometry experiment. In the western blot experiment, we performed it once, and examined n = 2 biological replicates per genotype. In the mass spectrometry experiment, we performed it once with n = 5 replicates.
Randomization	No randomization was used.
Blinding	We were blinded during the acquisition of imaging data, quantification, and were semi-blinded in behavior tests.

Reporting for specific materials, systems and methods

We require information from authors about some types of materials, experimental systems and methods used in many studies. Here, indicate whether each material, system or method listed is relevant to your study. If you are not sure if a list item applies to your research, read the appropriate section before selecting a response.

Materials & experimental systems

n/a	Involved in the study	
☐	☒	Antibodies
☒	☐	Eukaryotic cell lines
☒	☐	Palaeontology and archaeology
☐	☒	Animals and other organisms
☒	☐	Clinical data
☒	☐	Dual use research of concern

Methods

n/a	Involved in the study	
☒	☐	ChIP-seq
☒	☐	Flow cytometry
☒	☐	MRI-based neuroimaging

Antibodies

Antibodies used	The following primary antibodies were used for immunoblotting: 1:1,000 Rab35 (rabbit; Cell Signaling, 9690S), 1:10,000 Actin [C4] (mouse; Merck-Millipore, MAB1501), 1:10,000 GAPDH [6C5] (mouse; Merck-Millipore, MAB374), 1:1,000 Contactin-2/TAG-1 (goat; R&D systems, AF4439), 1:1,000 CHL1 (goat; R&D systems, AF2147), and 1:1,000 N-cadherin [32/N-cadherin] (rabbit; BD, 610920). The following primary antibodies were used for immunostaining: 1:100 NeuN [A60] (mouse; Merck-Millipore, MAB377), 1:200 GFAP

	(rabbit, Frontier Institute, GFAP-Rb-Af800), 1:200 b-galactosidase (mouse, Promega, Z3781), 1:100 Cux1 (rabbit; Santa Cruz, sc13024), 1:100 Ctip2 (rat; Abcam, ab18465), 1:300 phosphorylated neurofilament [SMI-31] (mouse; Covance, SMI-31R), 1:500 Tbr2 (rabbit; Abcam, ab23345), 1:300 Pax6 (rabbit; BioLegend, PRB-278P), 1:200 phospho-Histone H3 (Ser10) [RR002] (rabbit; Cell Signaling, 9701), and 1:100 Nestin [Rat-401] (mouse; Invitrogen, 14-5843-82). The following secondary antibodies were used for immunostaining: goat anti-rat Alexa Fluor-488, donkey anti-mouse Alexa Fluor-488, donkey anti-rabbit Alexa Fluor-488, donkey anti-mouse Alexa Fluor-594, and donkey anti-rabbit Alexa Fluor-594 (all antibodies are from Life Technologies).
Validation	Commercially available antibodies were validated for their applications respectively.

Animals and other research organisms

Policy information about studies involving animals; ARRIVE guidelines recommended for reporting animal research, and Sex and Gender in Research

Laboratory animals	We used C57BL/6 background wild type and knockout mice.<br>For behavioral analysis, 3-4 months Male mice were used. For immunoblot analysis, mixed gender were used in experiments with embryonic or P0 samples. Male adult control and cKO samples were used. For immunohistochemistry, 3-4 months mixed sex mice were used after comfirming that there are no gender differences. In experiments using embryos, we did not identified sex.
Wild animals	No wild animals were used in this study.
Reporting on sex	See above.
Field-collected samples	No field-collected samples were used in this study.
Ethics oversight	All animal procedures were approved by the Animal Care and Experimentation Committee of Gunma University.

Note that full information on the approval of the study protocol must also be provided in the manuscript.
